# Controlled Synthesis and Selective Adsorption Properties of Pr_2_CuO_4_ Nanosheets: a Discussion of Mechanism

**DOI:** 10.1186/s11671-018-2697-9

**Published:** 2018-09-05

**Authors:** Xuanwen Liu, Zhiyuan Ni, Chengzhi Xie, Renchao Wang, Rui Guo

**Affiliations:** 1 School of Materials Science and Engineering, Northeastern University, Shenyang, 110819 People’s Republic of China; 2 Key Laboratory of Nano-Materials and Photoelectric Catalysis of Qinhuangdao, School of Resources and Materials, Northeastern University at Qinhuangdao, Qinhuangdao, 066004 People’s Republic of China; 3 Tianjin Key Laboratory on Technologies Enabling Development of Clinical Therapeutics and Diagnostics (Theranostics), School of Pharmacy, Tianjin Medical University, Tianjin, 300070 People’s Republic of China

**Keywords:** Pr_2_CuO_4_, Coordination compound methods (CCMs), Selective adsorption, Malachite green(MG), Density functional theory (DFT)

## Abstract

Tetragonal-phase Pr_2_CuO_4_ nanosheets with a thickness of about 60 nm were synthesized using the coordination compound methods (CCMs), then used as highly efficient selective adsorbent towards malachite green (MG) in aqueous solutions. The Pr_2_CuO_4_ samples were characterized using X-ray diffraction (XRD), scanning electron microscopy (SEM), high-resolution transmission electron microscopy (HRTEM), X-ray photoelectron spectroscopy (XPS), UV-Vis diffuse reflectance spectrum (DRS), and standard Brunauer–Emmett–Teller (BET) methods. The maximum adsorption capacity (*Q*_*m*_) of as-prepared samples was determined by adsorption isotherms with different adsorbent doses (*m*) of 0.03–0.07 g at 298, 318, and 338 K based on the Langmuir model. When *m* < 0.03 g or > 0.07 g, effects of systemic mass loss and particle aggregation were discussed on the data deviation from the Langmuir model at 298 K. Based on the hydrogen bond and coordination bond, a possible mechanism of selective adsorption of MG by Pr_2_CuO_4_ is proposed, which was further verified by the adsorption experiments of CuO and Pr_2_O_3_ towards MG and competing-ion experiments. Finally, the theoretic studies were performed at DFT level to reveal the possible adsorption process.

## Background

Over the past few decades, dye-containing wastewaters discharged by industries are particularly dangerous pollutants because dyes, such as methyl orange (MO), methylene blue (MB), rhodamine B (RhB), malachite green (MG), and so on, are not biodegradable in human body [1–6]. Of these, MG as a commonly used dye has recently been used as a bactericidal agent for fish eggs [7, 8]. Therefore, it often appears in surface water with other wastewater together, posing a serious threat to human health [9, 10]. Consequently, many adsorbents such as the nano-oxide (i.e., ZnO and ZrO) [11–13], mesoporous materials (i.e., ordered mesoporous carbons and mesoporous poly(acrylic acid)/SiO_2_) [14, 15], and some metal-organic frameworks (MOFs) [1] have been reported working as the adsorption of MG. Flower-like ZnO, among these adsorbents, was reported with largest *Q*_*m*_ (maximum adsorption capacity) of 2587 mg/g. However, their adsorption capacities of MG are greatly reduced in real water because these adsorbents are easily covered by various organic compounds. Therefore, studies focusing on large-capacity and selective adsorbents are important for the adsorption of organic dyes [7, 8, 16, 17]. It is encouraging that rare earth cuprate exhibits high selective adsorption of MG with the largest special adsorption capacities (i.e., the *Q*_*m*_ of Dy_2_Cu_2_O_5_ is higher than 5.54 g/g) [17]; however, the mechanism is still not very clear.

This selective adsorption of rare earth cuprate should be studied based on the specific molecular structure of MG, which is different from other dyes. As reported by Y. Li et al. [4], MG has an isomer (leucomalachite green, LMG) in aqueous solution, containing coordinatable oxygen atoms. Therefore, we have proposed a mechanism based on coordination bonds during adsorption processes, as that in MOFs [1, 2, 17].

In this work, we provide a deeper insight into the deviation of the adsorption data from the Langmuir model for the adsorption process of MG on Pr_2_CuO_4_ adsorbent. Another goal is to explain the large selective adsorption of Ln–Cu–O compounds to MG and the possibility of multilayer adsorption mechanism. For the possibility of the formation of hydrogen bond and coordination bond in the adsorption process, theoretical studies were carried out at the DFT level.

There are very few reports on the chemical properties of Ln_2_CuO_4_-type rare earth cuprates, compared to the numerous catalysts and adsorbents of transition metal oxides and rare earth oxides [18–20]. To the best of our knowledge, this is the first report related to the adsorption mechanism of Pr_2_CuO_4_ towards MG, accompanied by a large *Q*_*m*_ value at room temperature.

## Methods/Experimental

### Materials

Cu(OAc)_2_·4H_2_O, Pr(NO_3_)_3_·5H_2_O, 3,4-pdc, and triethylamine were purchased from Sinopharm Chemical Reagent Co. Ltd. (Shanghai, China). Malachite green (MG) were bought from Aladdin Industrial Corporation, Shanghai. All reagents used in this study were of analytical grade and used without further treatment.

### Synthesis

The CCMs precursor [PrCu(3,4-pdc)_2_(OAc)(H_2_O)_2_] •10.5H_2_O was prepared according to our previous study [21, 22]. Cu(OAc)_2_·4H_2_O, Pr(NO_3_)_3_·5H_2_O, 3,4-pdc, and triethylamine with corresponding stoichiometric proportions were dissolved in a mixture of water-methanol at the volume ratio of 1:1. The solution was stirred for 3 h, then filtered off and allowed to stand until the formation of blue polycrystal. The obtained crystals were then calcined at different temperature for 1 h under N_2_ atmosphere to yield Pr_2_CuO_4_.

### Characterization

XRD patterns of as-prepared samples were obtained on a D/Max-RB X-ray diffractometer (Rigaku, Japan) using Cu *Kα* irradiation at a scan rate (*2θ*) of 0.05°/s from 10 to 90 °. Powder morphologies were characterized using SEM (Zeiss Supra 55, German) and HRTEM (FEI Tecnai F30, America). Selected area electron diffraction (SAED) pattern and high-angle annular dark-field (HAADF) imaging were acquired to measure the individual nanoparticles. The size distribution of as-prepared Pr_2_CuO_4_ is detected using a laser granularity meter (Mastersizer 2000, England). The specific surface areas of the as-prepared samples were measured by N_2_ adsorption/desorption experiments using a Builder SSA-4300. DRS was measured by a UV-Visible (PERSEE T9, China) spectrophotometry with BaSO_4_ as the reference sample. Oxidation states of the elements of the catalyst were obtained by high-resolution X-ray photoelectron spectroscopy (XPS) on a PHI 5000 C ESCA System (Japan) with Mg *K* source operating at 14.0 kV and 25 mA.

### Adsorption Experiments

The adsorption of MG from aqueous solution was conducted in batch experiments using the Pr_2_CuO_4_ particles as adsorbents with an overhead stirrer at 100 rpm. Various adsorbent doses (0.03–0.07 g) were added to 1000 mL of 0.1 g/L MG aqueous solution. Once the equilibrium was established, the solution was filtered and the filtrate was analyzed using an UV-Visible (RF 5301) to determine the residual concentration of MG. The adsorbed amount was calculated using Eq. (1).

1 $$ {q}_e=\frac{\left({C}_0-{C}_e\right)\times V}{m} $$

where *q*_*e*_ (mg/g) is the adsorption capacity at the equilibrium concentration and *C*_*0*_ (mg/L) and *C*_*e*_ (mg/L) are the initial and equilibrium concentrations of MG in the aqueous solution, respectively. *V* (L) is the initial solution volume and *m* (g) represents the mass of the used dry adsorbent.

Langmuir equation and Freundlich equation in linear form are expressed as

2 $$ \frac{1}{q_e}=\frac{1}{K^{\theta }{Q}_m}\times \frac{1}{C_e}+\frac{1}{Q_m} $$

3 $$ \ln {q}_e=\ln {K}_F+\frac{1}{n}\ln {C}_e $$

where *K*^*θ*^ is the Langmuir constants, and *K*_*F*_ and *n* are Freundlich constants. The kinetic characteristics of the adsorption process prior to equilibrium were analyzed using the time response of the isothermal adsorption experiments as described above. For comparison, the competing-ion (related to O–Pr and O–Cu coordination bonds) experiments, including methyl orange (MO) and rhodamine B (RhB), were performed under the same conditions. The initial concentration of the competing ion was set to 0.02 g/L.

### Theoretical Studies

The DFT calculations were performed using a DMol^3^ package of Materials Studio (version 7.1). All core electrons were calculated using the effective core potentials to reduce computational costs. The double-numeric quality basis set with polarization functions (DNP) was used for all atoms in the system. Geometry optimization of the surface of the adsorbents was implemented by using PerdewWang (PW91) exchange-correlation functional in the generalized gradient approximation (GGA). The cut-off energy of the plane wave functions and self-consistent field (SCF) tolerance were set to 340 eV and 1 × 10^− 6^ eV/atom, respectively. All the calculations were performed in reciprocal space.

## Results and Discussion

### Characterizations

**Fig. 1:**
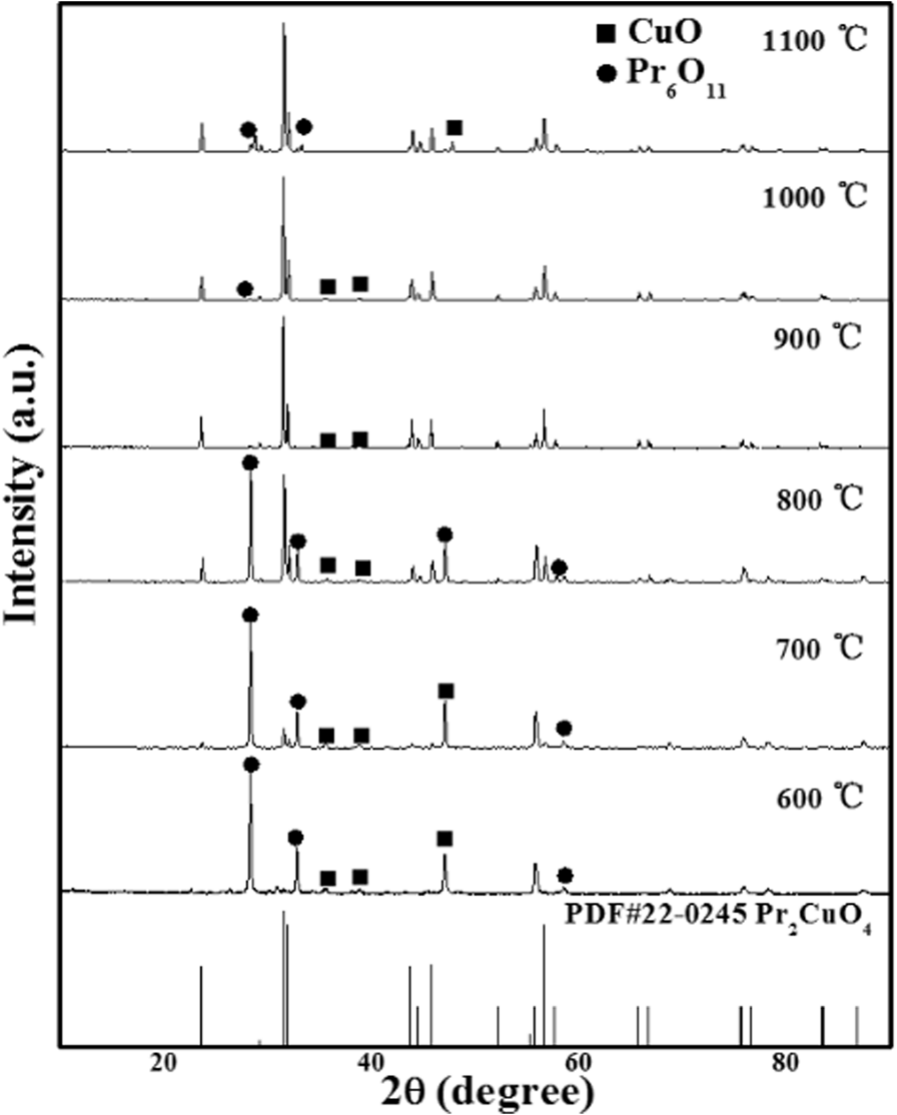
XRD patterns of Pr_2_CuO_4_ synthesized at 600–1100 °C and PDF# 22-0245

The XRD patterns of the Pr_2_CuO_4_ samples synthesized at different temperatures are shown in Fig. 1. At 600–700 °C, the crystallite of Pr_2_O_3_ and CuO appears with no Pr_2_CuO_4_, suggesting the temperature is too low to active Pr_2_O_3_ and CuO to form Pr_2_CuO_4_ [21]. At 800 °C, some characteristic peaks related to tetragonal-phase Pr_2_CuO_4_ (PDF # 22-0245) could be observed at Bragg angles of 23.5 ° and 31.5 °; however, there is still a large amount of Pr_2_O_3_ and CuO in the sample. At 900 °C, more Pr_2_O_3_ and CuO are reacted to form Pr_2_CuO_4_ with a slight amount of CuO residual. The diffraction peaks are sharp and intense, indicating high crystallinity of the sample. No other impurity peaks are observed, confirming the high purity of Pr_2_CuO_4_. As the temperature increases up to 1000 °C, the sample still maintains a perfect purity. When the temperature exceeds 1100 °C, more impurity phase of CuO appears obviously due to the decomposition of the sample. Therefore, all the samples studied in adsorption experiments were synthesized at 900 °C.

**Fig. 2:**
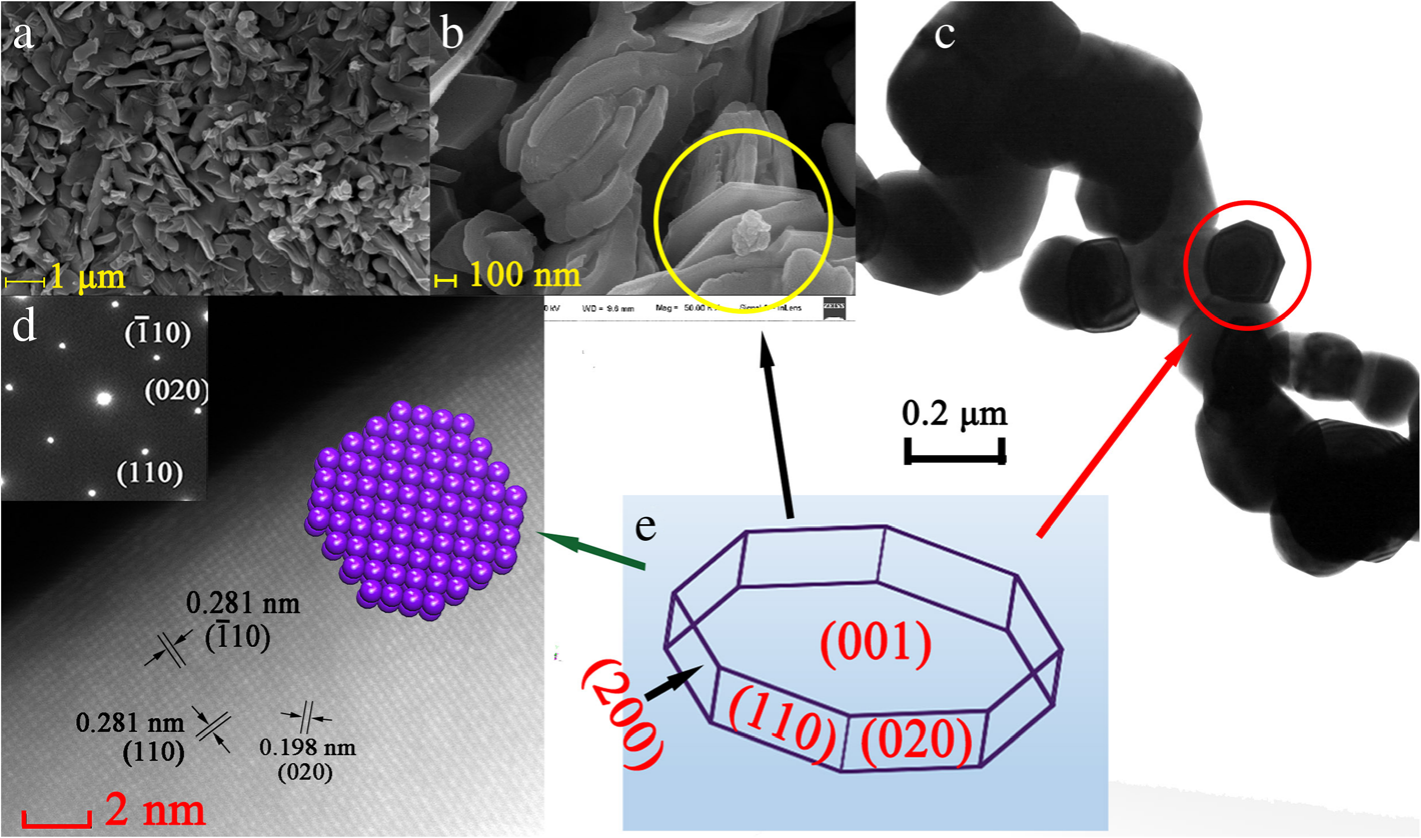
SEM image of Pr_2_CuO_4_ sample prepared at 900 °C (**a**, **b**), TEM (**c**), SAED and HAFFD images (**d**), and octagonal structure diagram (**e**)

Figure 2a, b shows the SEM images of Pr_2_CuO_4_ particles prepared at 900 °C. It could be seen that the Pr_2_CuO_4_ particles are well dispersed nanosheets, having an average thickness of about 60 nm. Most nanosheets are stacked together like lava, but the layer structure is still clearly observed. Few well-crystallized nanosheets show regular octagonal-sheet structure (in the yellow circle in Fig. 2b). The nanosheets are interconnected to build a three-dimensional hole, which is large enough for organic molecules to pass through, suggesting a perfect nature as adsorbents. As described in Ref. [20], the coordination precursor is continuously melted at the temperature above 300 °C to form small mobile phases, then solidified into oxides, and eventually broken into overlapping sheets (Fig. 2b). Since the metal ions are uniformly distributed in the coordination precursor, the product consists of polycrystalline particles by calcination at lower temperatures (< 900 °C, compared to solid-state sintering method).

The detailed structure of Pr_2_CuO_4_ is further revealed by high-resolution TEM images, SAED, and HAADF. Figure 2c shows the octagonal structure again (in red circle), which is consistent with SEM images. The HAADF image in Fig. 2d shows that Pr_2_CuO_4_ sample displays clear lattice spacings, indicating its single-crystallinity. The lattice plane spacing of 0.281, 0.281, and 0.198 nm match well with (− 110), (020), and (110) planes of tetragonal Pr_2_CuO_4_, respectively. A schematic diagram of the octagonal flaky structures in Fig. 2b, c is sketched in Fig. 2e and the facet index of the polyhedral sides are speculated by corresponding dihedral angles and XRD results. First, two crystal facet indices of the sides of the octagonal flaky are found to be (110) and (020) (Fig. 2d). Secondly, considering that the dihedral angle of the adjacent side of the octagon is approximately equal to 45 ° and the observed crystal plane indexed in Fig. 1, (200) crystal plane is deduced to be one side. Finally, considering that the upper surface is perpendicular to the side surface, the crystal facet index of the upper surface is determined to be (001). Since the thickness of the octagonal sheet is small, the X-ray diffraction intensity of {006} must be weak, as shown in Fig. 1, which indirectly supports the above assumptions. Therefore, it is believed that the as-prepared sample are likely surrounded by {110}, {020}, {200}, and {001}. Considering that the (001) plane has the largest exposed area, the (001) crystal face is selected as the adsorption surface in the DFT modeling.

**Fig. 3:**
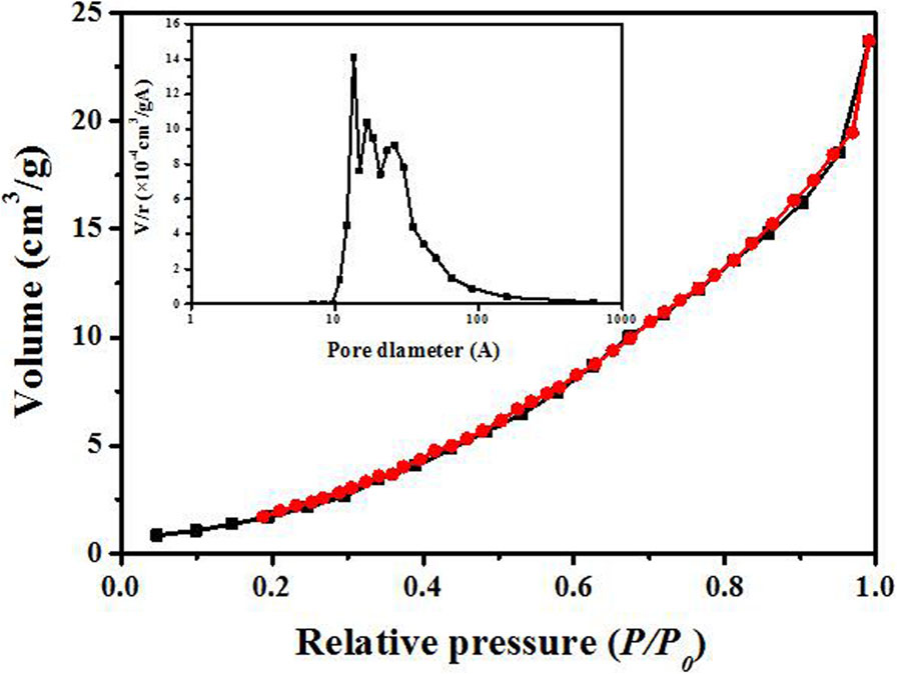
Nitrogen adsorption–desorption isotherms and the corresponding pore size distributions of Pr_2_CuO_4_ (the black line for the adsorption branch and the red line for the desorption branch)

Figure 3 shows the nitrogen adsorption–desorption isotherms and the corresponding pore size distribution of Pr_2_CuO_4_ adsorbent. It could be seen that the isotherm shows a type III isotherm according to the IUPAC classification, which is convex to the *p/p*_*0*_ axis over its entire range without a clear point to determine the beginning of multilayer adsorption [23, 24]. No obvious hysteresis loop is observed, suggesting a weak N_2_–Pr_2_CuO_4_ interaction. Moreover, its specific surface area is calculated to be 11.6 m^2^/g with pore sizes of 10–100 Å according to Brunauer–Emmet–Teller (BET) method, suggesting a very narrow spacing between the particles, which is consistent with SEM results.

**Fig. 4:**
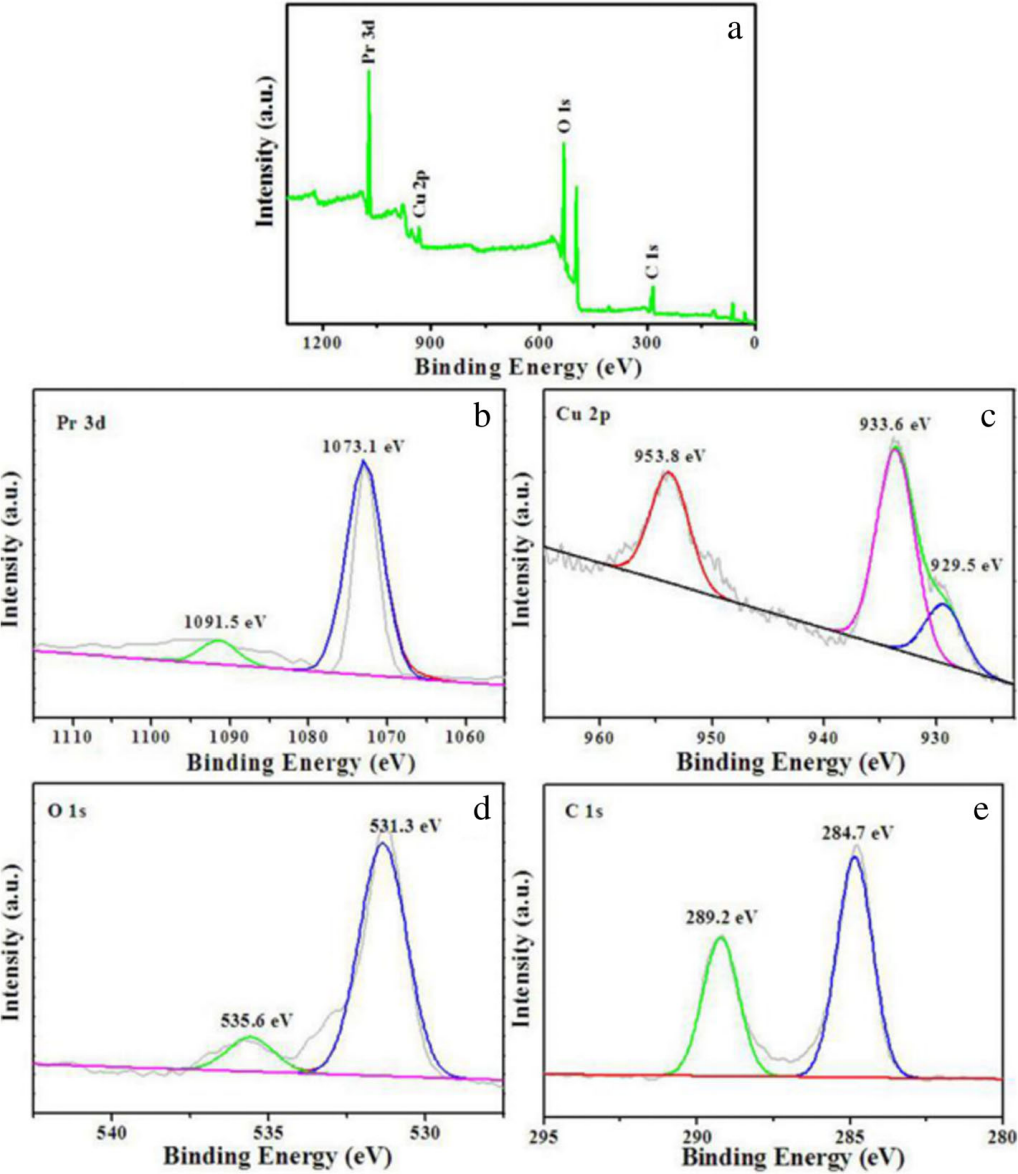
Total XPS spectra (**a**) and high-resolution XPS spectra of Pr 3d (**b**), Cu 2p (**c**), O 1 s (**d**), and C 1 s (**e**) of Pr_2_CuO_4_

The surface chemical composition and elemental states of Pr_2_CuO_4_ adsorbents are investigated by XPS. Figure 4a presents the XPS survey spectrum, showing that the sample contains Pr, Cu, O, and C elements. The high-resolution XPS spectra of Pr, Cu, O, and C were preciously deconvoluted considering spin-orbit coupling. The high-resolution XPS spectra of Pr 3d are shown in Fig. 4b. The peaks of 3d_5/2_ and 3d_3/2_ observed at 1073.1 and 1091.5 eV respectively confirm the presence of the chemical equivalent Pr ion with a formal charge of + 3 [25–27]. As shown in Fig. 4c, the Cu 2p XPS spectrum shows the core level of Cu 2p spectral region with one spin-orbit doublet. The main peaks represent Cu 2p_1/2_ at 953.8 eV and Cu 2p_3/2_ at 933.6 eV with an energy difference of about 20 eV, which could be attributed to Cu ion in CuO_4_ group with a formal charge of + 2 [28]. Meanwhile, a little peak observed at 929.5 eV could be attributed to the satellite peak of Cu 2p, which is possibly caused by the Cu ions with the lower-symmetric coordination environment in the adsorbent surface. Figure 4d shows two different valences of O at 531.3 eV and 535.6 eV (more positive), respectively, indicating that there are two kinds of non-equivalent O atoms. The peak centered at 531.3 eV represents the O atom surrounded by two Cu atoms and four Pr atoms in the CuO_2_ layer of Pr_2_CuO_4_ lattice, while the peak at 535.6 eV is assigned to the O atom coordinated with four Pr atom in the Pr_2_O_2_ layer of Pr_2_CuO_4_ lattice [29]. In Fig. 4e, the binding energy of adventitious carbon (284.7 eV) is applied for charge correction. However, the peak at 289.5 eV can be attributed to C–O species, suggesting the presence of C residue, which could be seen as one of the characteristics of CCMs.

**Fig. 5:**
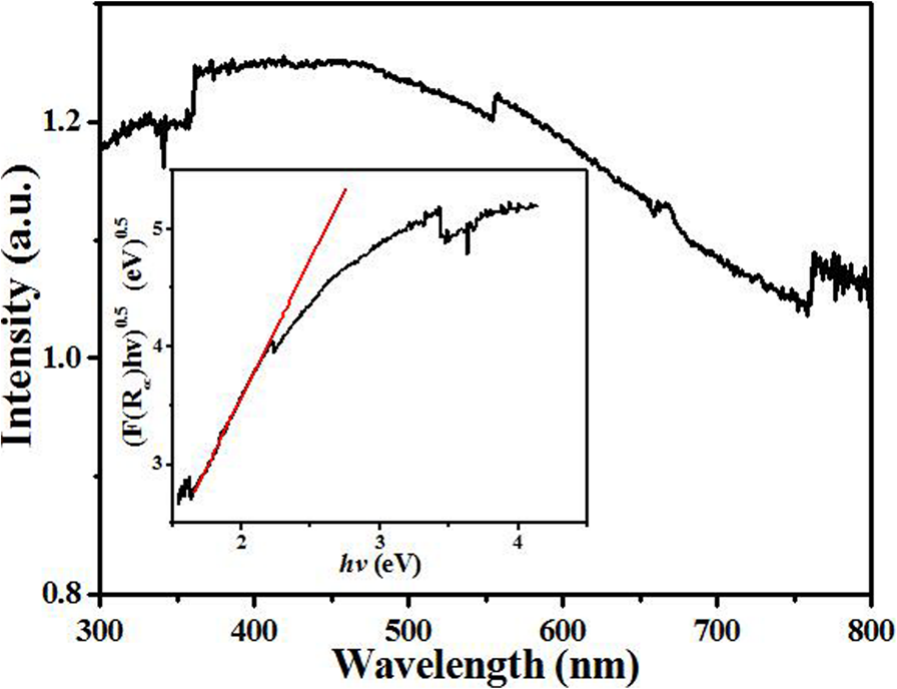
UV-Vis diffuse reflectance spectra of Pr_2_CuO_4_ samples and the determined direct interband transition energies

Figure 5 shows the UV-Vis absorption spectra of Pr2CuO4. The strong and broad spectrum absorption band from 750 to 300 nm could be clearly observed due to the strong d–d electron transitions and charge transfer transitions of Cu–O and Pr–O [30, 31]. Thus, the sample appears dark blue. The strong absorption of light makes Pr2CuO4 a potential photocatalyst, but no photocatalytic phenomena have been observed. It indirectly means that the recombination of photogenerated electron-hole pairs of Pr2CuO4 is intensive. The direct interband energy gap is calculated to be 0.51 eV (the inset in Fig. 5), revealing that photo-generated electron could be easily relaxed by lattice vibration. Thus, the photocatalytic properties of Pr2CuO4 are not observed.

### Maximum Adsorption Capacity and Mechanism

**Fig. 6:**
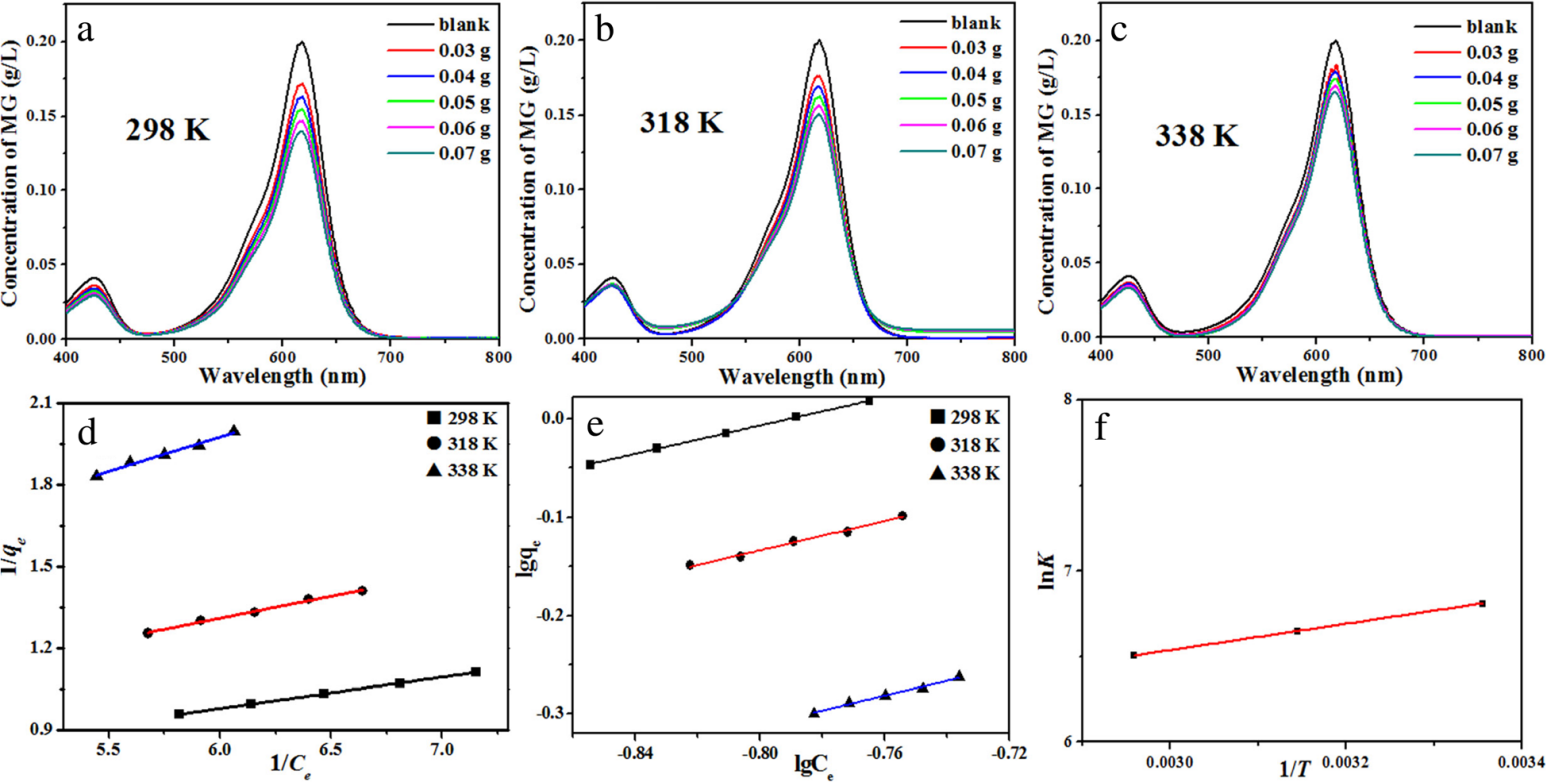
Varieties MG equilibria concentrations in the maximum adsorption experiment of CCMs at 298 (**a**), 318 (**b**), and 338 K (**c**) and the corresponding fitting lines according to Eqs. (2), (3), and (4) are depicted in (**d**, **e**, and **f**), respectively

The adsorption capacities of Pr_2_CuO_4_ are evaluated by equilibrium adsorption experiments at 298, 318, and 338 K, as shown in Fig. 6a–c. When the adsorption reactions reached equilibrium at 298 K, the equilibrium concentration of MG significantly decreases with increasing the adsorbent dosage (Fig. 6a). As the temperature rises, the equilibrium concentration of MG is gradually increased in the case of the same dose of adsorbent, indicating that the temperature rise positively affects the desorption of MG (Fig. 6b, c). According to the Langmuir model (Eq. 2) and the Freundlich model (Eq. 3) [32], the data of Fig. 6a–c are depicted in Fig. 6d, e. The values of the related parameters and corresponding *R*^*2*^ are listed in Table 1. The results show that the isotherms follow the Langmuir model better with a higher value of *R*^*2*^ than the Freundlich model. Therefore, the *Q*_*m*_ of the Pr_2_CuO_4_ adsorbent calculated according to the Langmuir model reaches as high as 3.52 g/g at 298 K. For comparison, the maximum MG adsorption capacities of some selected adsorbents are summarized in Table 2. To the best of our knowledge, the *Q*_*m*_ of the Pr_2_CuO_4_ to MG is only slightly lower than that of the analog Sm_2_CuO_4_ but much larger than physical adsorbents such as bamboo-based activated carbon, suggesting that the adsorption mechanism is likely to be different from ordinary physical adsorption. The *Q*_*m*_ of Pr_2_CuO_4_ decreases to 2.17 g/g with the temperature rising to 338 K. Simultaneously, the equilibrium constant (*K*^*θ*^) drops from 906 to 667 L·mol^− 1^ as the temperature rises from 298 to 338 K, implying that the adsorption process is exothermic [33].

**Table 1:** Partial fitting results obtained from the maximum adsorption capacity experiments of CCMs

*T*(K)	Langmuir			Freundlich		
	*Q*_*m*_ *(g/g)*	*K*^*θ*^(L/mol)	*R* ^*2*^	*K*_*F*_(L/mol)	*1/n*	*R* ^*2*^
298	3.52	906	0.9968	1358	0.722	0.9886
318	2.93	771	0.9886	1059	0.746	0.9894
338	2.17	667	0.9849	720	0.759	0.9648

**Table 2:** Comparison of adsorption capacities of different adsorbents for MG at 298 K

Adsorbent	Adsorption capacity (mg/g)	Data resource
Bamboo-based activated carbon	263.58	[36]
ZnO-activated carbon	322.58	[9]
Ordered mesoporous carbons	354.5	[12]
Cellulose	458.72	[37]
Porous C–ZrO_2_ composite	2500	[11]
ZnO flower-like architectures	2587.0	[10]
Sm_2_CuO_4_	7180	[38]
This work	3520	

The thermodynamic parameters are fitted from the data in Table 1 according to Eq. (4) and the results are shown in Fig. 6f [34]:

4 $$ \ln {K}^{\uptheta}=\frac{-{\Delta}_r{G_m}^{\theta }}{RT}=-\frac{\Delta_r{H_m}^{\theta }}{R}\times \frac{1}{T}+\frac{\Delta_r{S_m}^{\theta }}{R} $$

where *Δ*_*r*_*G*_*m*_^*θ*^, *Δ*_*r*_*H*_*m*_^*θ*^, and *Δ*_*r*_*S*_*m*_^*θ*^ are the standard Gibbs free energy change, standard enthalpy change, and standard entropy change for adsorption of 1 mol MG, respectively.

*Δ*_*r*_*G*_*m*_^*θ*^, *Δ*_*r*_*H*_*m*_^*θ*^, and *Δ*_*r*_*S*_*m*_^*θ*^ are calculated to be − 16.9 kJ/mol, − 6.41 kJ/mol, and 35.1 J/mol·K, respectively. The negative value of *Δ*_*r*_*G*_*m*_^*θ*^ indicates that the adsorption reaction is spontaneous. The negative value of *Δ*_*r*_*H*_*m*_^*θ*^ further interprets the decrease of the equilibrium constant with increasing temperature. The positive value of *Δ*_*r*_*S*_*m*_^*θ*^ might imply that the adsorbent surface is initially covered by water molecules and the adsorbed MG molecule occupies a large area on the surface [33].

### Analysis of Deviation

**Fig. 7:**
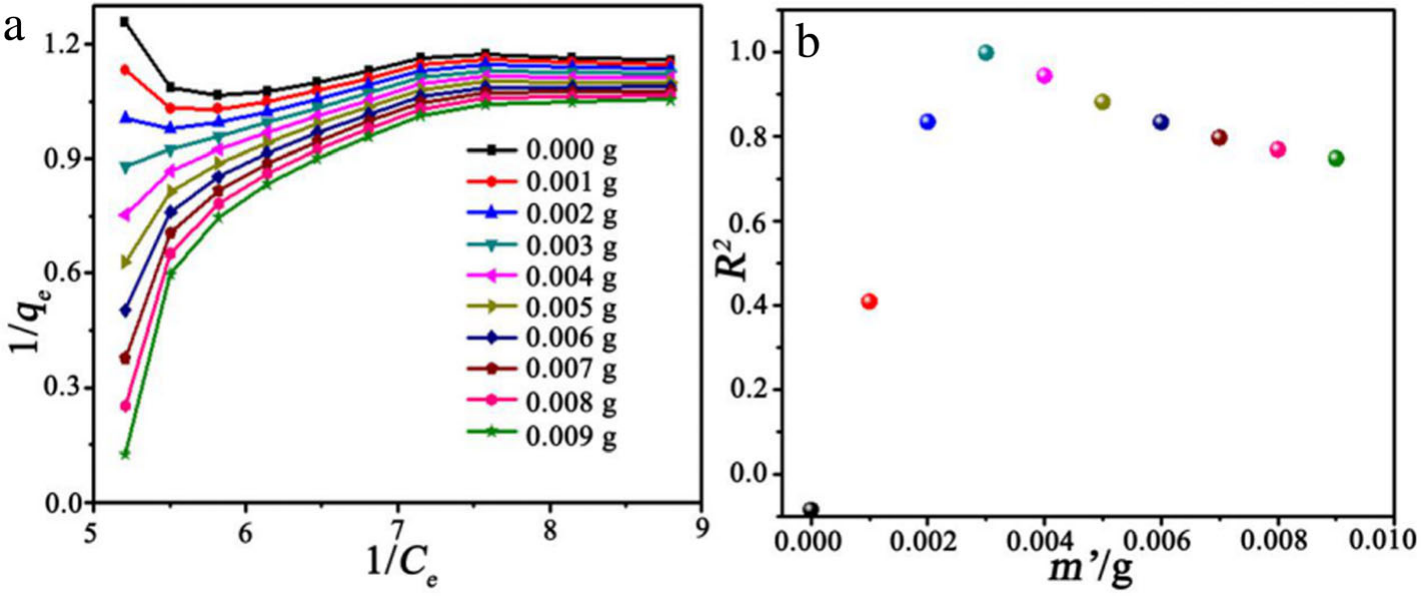
The plot of 1/*q*_*e*_ versus 1/*c*_*e*_ according to Eq. 2 (where, *m’* = *0*) with *q*_*e*_ modified by Eq. 5 with different value of *m’* ranging from 0.000 to 0.009 g at 298 K (**a**). The corresponding *R*^*2*^ as a function of *m’* (**b**)

The plot of 1/*q*_*e*_ versus 1/*c*_*e*_ according to Eq. 2 with *q*_*e*_ modified by Eq. 5 with *m’* ranging from 0 to 0.009 g at 298 K is depicted in Fig. 7a. It is clearly observed that the plot, when *m’* = 0, shows a significant deviation from the Langmuir model (0.01 g < *m* < 0.03 g and 0.07 g < *m* < 0.10 g). Therefore, the plot of 1/*q*_*e*_ versus 1/*c*_*e*_ needs to be calibrated by introducing a factor *m’* according to Eq. 5. As shown in Fig. 7b, when 0.001 g < *m’* < 0.003 g, *R*^*2*^ increases steadily with *m’*, while *R*^*2*^ decreases quickly when *m’* exceeds 0.004 g. Therefore, the optimal value of *m’* is 0.003 g, indicating that there may be a systematic error for some reasons, such as the agglomeration of adsorbent particles. The agglomeration of the adsorbent particles may be due to the increase in viscosity of the adsorbent particles after the adsorption of MG molecules. The corresponding mechanism and process are demonstrated in the next section.

5 $$ {q}_e=\frac{\left({C}_0-{C}_e\right)\times V}{m-{m}^{\hbox{'}}} $$

**Fig. 8:**
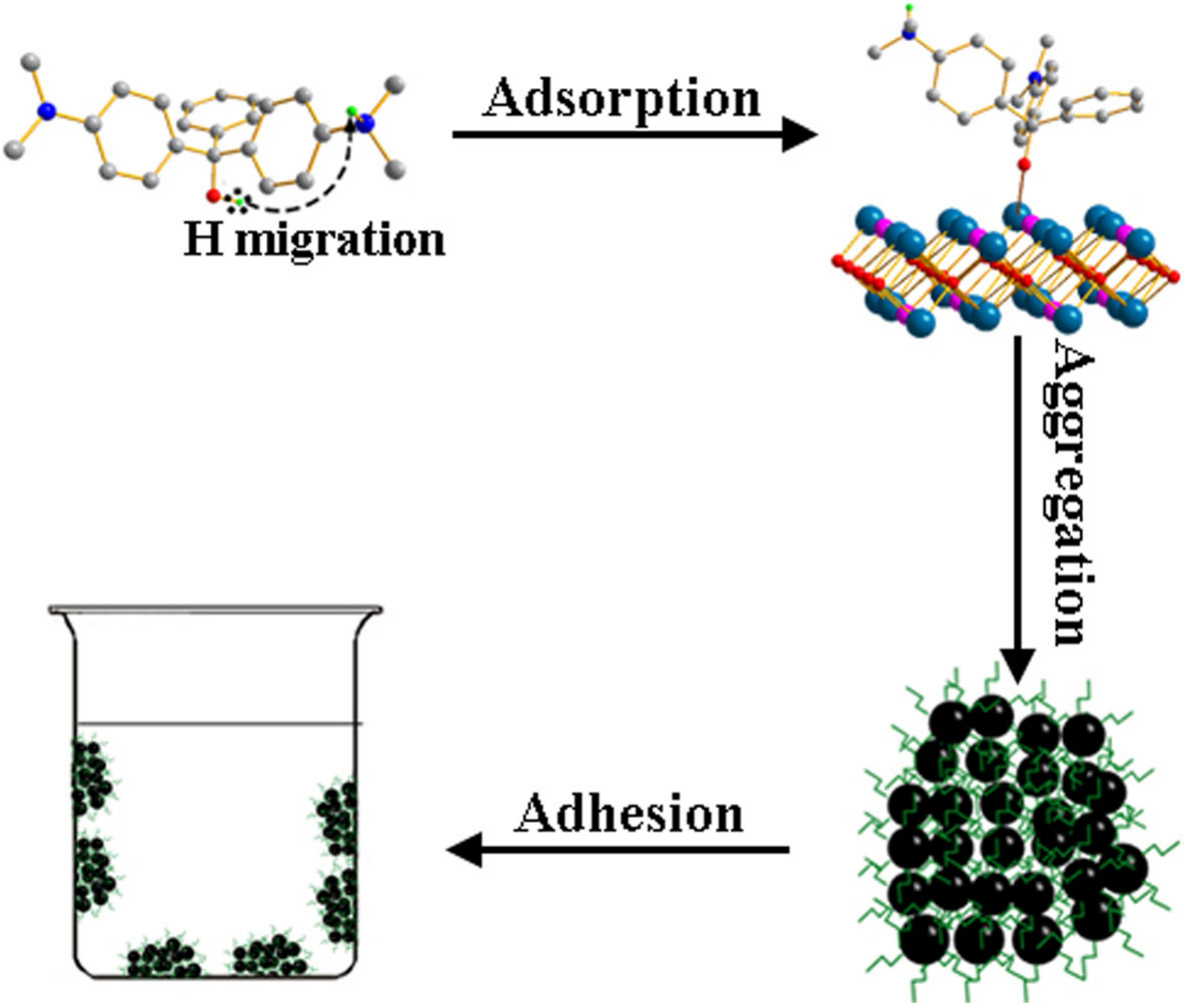
The schematic diagram for the aggregating process of Pr_2_CuO_4_ particles with huge *Q*_*m*_ in water and water/ethanol mixture

The aforementioned correction method can explain the deviation well in the case of *m* < 0.04 g, but when *m* > 0.07 g, it becomes very difficult to explain the deviation. The addition of higher-order term of *m* to *m’* is an option to explain the deviation for *m* > 0.07 g; however, its physical meaning becomes ambiguous. Another method is to adopt the multi-layer adsorption theory. An obvious experimental phenomenon, the appearance of dark blue spots on the container wall, supports this theory. It suggests the possibility of the aggregation of adsorbent particles. The possibility might be due to the rearrangement of H atoms during the adsorption of MG [4], as shown in Fig. 8. The migration of hydrogen atoms produces O^−^ ion and NH_3_^+^ ion, generating a dipolar MG. Since the polarization of MG molecules greatly increases the intermolecular interaction, the adsorbent particles with adsorbed MG molecules tend to aggregate together and attach to the walls of the container. An aqueous/ethanol mixture solution at 1:1 volume ratio was used as the solution, and the above isotherm experiment was repeated at 298 K. The degree of aggregation decreased obviously in the aqueous/ethanol mixture solution, which could be explained by the depolarization of MG molecule in a weakly polar solvent.

### Theoretical Analysis at DFT Level

**Fig. 9:**
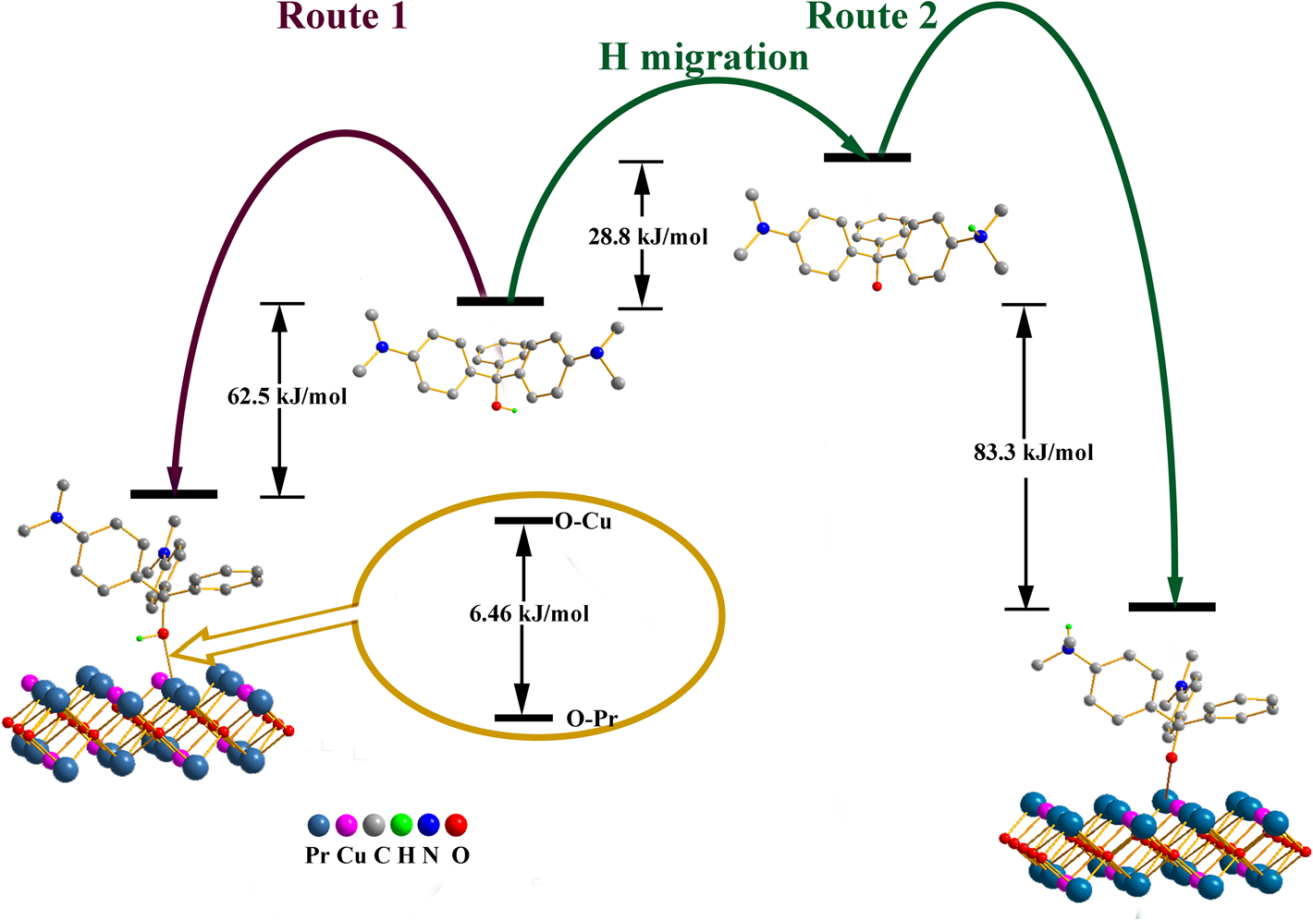
The schematic diagram for the energy changes of route 1 and 2 based on DFT studies

**Fig. 10:**
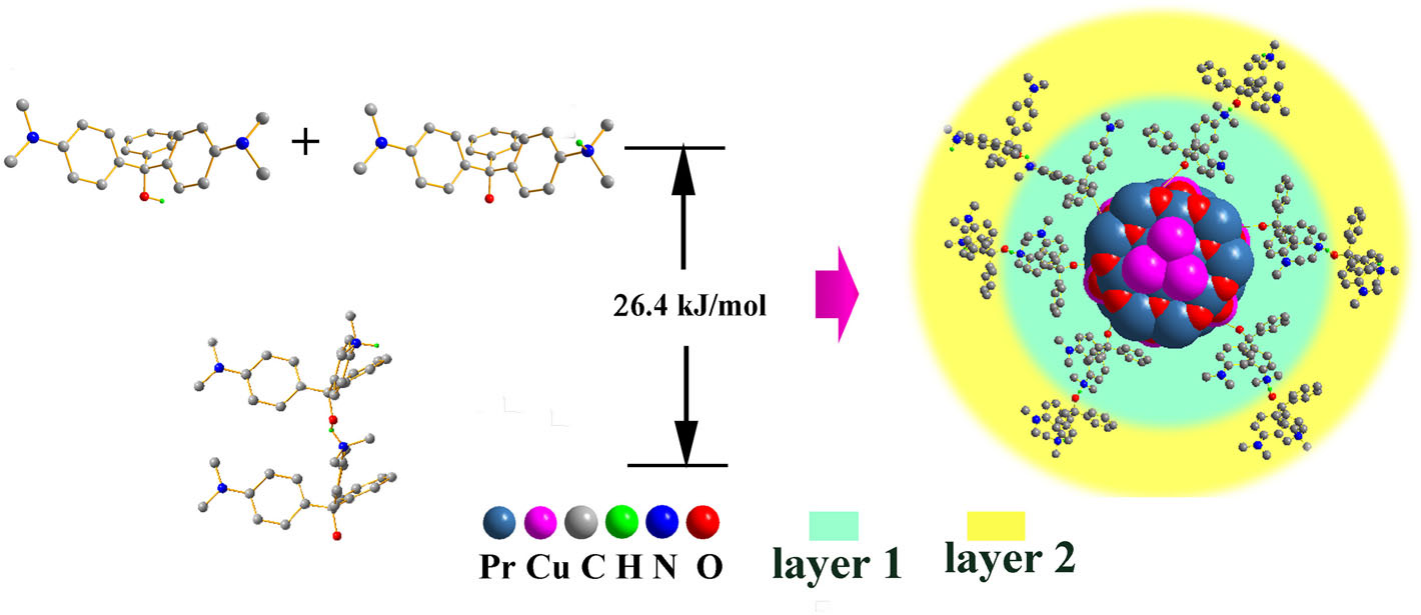
The schematic diagram for the energy changes of multilayer adsorption by H-bond between dipole MG molecules

The above assumptions are further analyzed by the DFT method. As reported by Li et al. [4], the isomer of MG (in Fig. 9) contains coordinatable oxygen atoms, which has the ability to connect with Cu and Pr atoms of the adsorbent. This mode is described as route 1 in Fig. 9. The adsorption energy of route 1 is calculated to be 62.5 kJ/mol based on the O–Pr coordination bond at the DFT level, which is 6.46 eV/mol larger than that of O–Cu. Based on this, route 2 is represented by two stages: (i) the H atom of the MG molecule migrates from the hydroxyl group to the amino group with an energy rise of 28.8 kJ/mol and a activation energy of 309.8 kJ/mol, close to the bond energy of O–H. However, the ionization of O enhances the adsorption strength by a stronger O–Pr coordination bond with a larger adsorption energy of 83.3 kJ/mol. The product of route 2 is more stable by 20.8 kJ/mol compared to route 1. The length of O_MG_–Pr coordination bond is calculated to be 2.99 Å, slightly larger than those in Cu–Pr coordination complexes (i.e., 2.36 Å in CCDC: 1524771), suggesting a strong interaction between Pr and O_MG_. (ii) The ionized MG molecule could induce the polarization of adjacent MG molecules, which increases the electrostatic interaction between the MG molecules and further form H···N bonds. Consequently, a multilayer adsorption with an energy drop of 26.4 kJ/mol is obtained. The value of route 2 is more consistent with the above thermodynamic results, implying route 2 is more reliable. After the formation of hydrogen bonds (Fig. 10), the bond lengths of O–H is stretched to 1.07 Å, 0.10 Å longer than that in free MG molecule. The H bond length of H…N is about 1.60 Å, implying that covalent interaction between MG molecules plays a key role in the formation of hydrogen bonds. In route 2, a large number of ionized MG molecules adsorbed on the surface of Pr_2_CuO_4_ is electrostatically viscous, which might explain the agglomeration of adsorbent particles during the adsorption process (Fig. 8). Therefore, the multilayer adsorption route might be the major mode, which could explain the large *Q*_*m*_ of Pr_2_CuO_4_ well. The above mechanism is similar to that of the pH-dependent adsorption of ionizable compounds, reported by Tang [35].

**Fig. 11:**
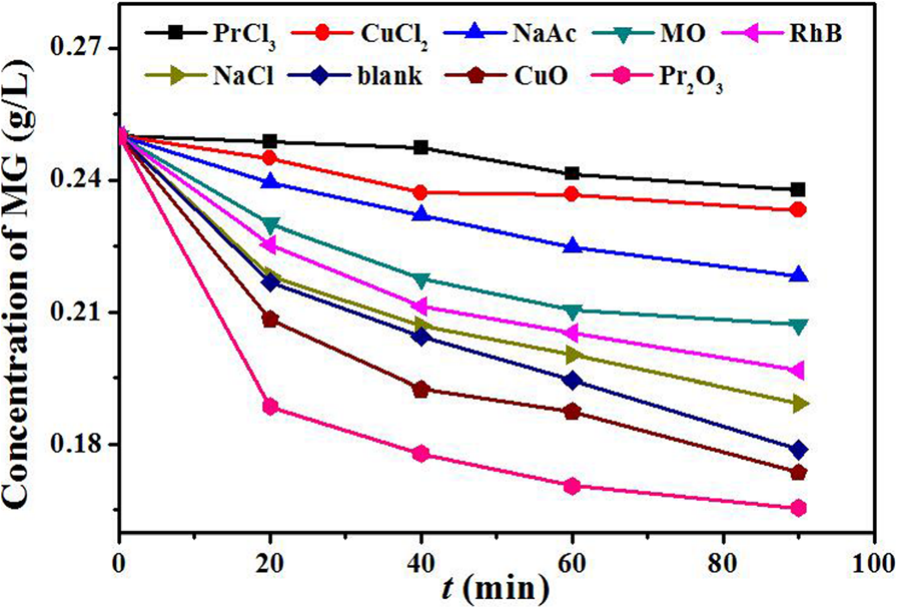
Effects of competitive ions, dyes, CuO, and Pr_2_O_3_ on the adsorption of Pr_2_CuO_4_ for MG

To gain a better understanding of the adsorption mechanisms, isothermal adsorption experiments with different competitive ions, dyes, and oxides were also carried out and the data are depicted in Fig. 11. Dyes such as MO and RhB have little effect on the adsorption process, suggesting Pr_2_CuO_4_ is a selective adsorbent. The ions (Cl^−^ anions and Na^+^ cations) also showed a little effect on the adsorption process, suggesting that the selective adsorption is different from electrostatic adsorption. The effect of OAc^−^ is stronger than that of Cl^−^, due in part to the formation of O–Cu and O–Pr coordination bonds. Similarly, Cu^2+^ and Pr^3+^ could effectively block the adsorption of MG through coordination bonds. Meanwhile, CuO and Pr_2_O_3_ significantly increase the adsorption capacity, indicating that they might have the same adsorption mechanism as Pr_2_CuO_4_. These experimental results are consistent with the DFT analysis, which further supports the view of coordination adsorption.

## Conclusions

Pr_2_CuO_4_ adsorbents were successfully prepared via CCMs with a large *Q*_*m*_ of 3.52 g/g at 298 K. The deviation of the adsorption data from the Langmuir model is due to the systematic mass loss of 0.003 g, when *m* < 0.04. When *m* > 0.07 g, the effect of agglomeration of particles on the adsorption capacity could not be ignored. The large adsorption capacity of Pr_2_CuO_4_ adsorbent was discussed according to multilayer adsorption model: (i) the H atom of the MG molecule migrates from the hydroxyl group to the amino group to enhance the adsorption strength, with the adsorption energy of 83.3 kJ/mol. (ii) The polarized MG molecules are bound to each other by hydrogen bond during multilayer adsorption process with an energy drop of 26.4 kJ/mol. In addition, this multilayer adsorption mechanism was confirmed by the DFT studies and competing-ion experiments.

## Abbreviations

3,4-pdc: 3,4-pyridinedicarboxylic acid
CCMs: Coordination compound methods
HAADF: High-angle annular dark-field
MG: Malachite green
MOFs: Metal-organic frameworks
Q_m_: The maximum adsorption capacity
RhB: Rhodamine B
SAED: Selected area electron diffraction

## References

[1] Ting H, Chi HY, Lam CH, Chan KY, Kang DY (2017). High-permeance metal-organic framework-based membrane adsorber for the removal of dye molecules in aqueous phase. Environ Sci Nano.

[2] Zhao QH, Xing YX, Liu ZL, Jin OY, Du CF (2018). Synthesis and characterization of modified BiOCl and their application in adsorption of low-concentration dyes from aqueous solution. Nanoscale Res Lett.

[3] Pandey S (2017). A comprehensive review on recent developments in bentonite-based materials used as adsorbents for wastewater treatment. J Mol Liq.

[4] Li Y, Gao ZQ, Ji YF (2015). Photodegradation of malachite green under simulated and natural irradiation: kinetics, products, and pathways. J Hazard Mater.

[5] Trofymchuk I, Roik N, Belyakova L (2017). Structural variety and adsorptive properties of mesoporous silicas with immobilized oligosaccharide groups. Nanoscale Res Lett.

[6] Chen Y, Zhang YF, Luo LJ, Shi YY, Wang S, Li LX, Long YJ, Jiang FZ (2018). A novel templated synthesis of C/N-doped beta-Bi_2_O_3_ nanosheets for synergistic rapid removal of 17 alpha-ethynylestradiol by adsorption and photocatalytic degradation. Ceram Int.

[7] Fallah AA, Barani A (2014). Determination of malachite green residues in farmed rainbow trout inIran. Food Control.

[8] Meyer FP, Jorgenson TA (1983). Teratological and other effects of malachite green on development of rainbow trout and rabbits. Trans Am Fish Soc.

[9] Ding B, Cheng Y, Wu J, Wu XM, Zhang HM, Luo Y, Shi XF, Wu XX, Huo JZ, Liu YY (2017). A unique multifunctional cluster-based nano-porous terbium organic material: real-time detection of benzaldehyde, visually luminescent sensor for nitrite and selective high capacity capture of Congo Red. Dyes Pigments.

[10] Li HZ, Fang XT, Ma S, Niu YF, Zhao XL, Xu JQ, Duan ZM (2017). A two-dimensional porous framework: solvent-induced structural transformation and selective adsorption towards malachite green. Dalton Trans.

[11] Ghaedi M, Ansari A, Habibi MH, Asghari AR (2014). Removal of malachite green from aqueous solution by zinc oxide nanoparticle loaded on activated carbon: kinetics and isotherm study. J Ind Eng Chem.

[12] Pei CJ, Han GP, Zhao Y (2016). Superior adsorption performance for triphenylmethane dyes on 3D architectures assembled by ZnO nanosheets as thin as similar to 1.5 nm. J Hazard Mater.

[13] Deshpande PA, Polisetti S, Madras G (2011). Rapid synthesis of ultrahigh adsorption capacity zirconia by a solution combustion technique. Langmuir.

[14] Tian Y, Liu P, Wang XF, Lin HS (2011). Adsorption of malachite green from aqueous solutions onto ordered mesoporous carbons. Chem Eng J.

[15] Xu R, Jia M, Zhang YL, Li FT (2012). Sorption of malachite green on vinyl-modified mesoporous poly(acrylic acid)/SiO_2_ composite nanofiber membranes. Microporous Mesoporous Mater.

[16] Giri S, Das R, Westhuyzen C, Maity A (2017). An efficient selective reduction of nitroarenes catalyzed by reusable silver-adsorbed waste nanocomposite. Appl Catal B Environ.

[17] Liu XW, You JH, Wang RC, Ni ZY, Han F, Jin J, Ye ZQ, Fang ZF, Guo R (2017). Synthesis and absorption properties of hollow-spherical Dy_2_Cu_2_O_5_ via a coordination compound method with [DyCu(3,4-pdc)_2_(OAc)(H_2_O)_2_]•10.5H_2_O precursor. Sci Rep.

[18] Yang S, Lee H (2017). Determining the catalytic activity of transition metal-doped TiO_2_ nanoparticles using surface spectroscopic analysis. Nanoscale Res Lett.

[19] Li CH, Wei RX, Xu YM, Sun AL, Wei LH (2014). Synthesis of hexagonal and triangular Fe_3_O_4_ nanosheets via seed-mediated solvothermal growth. Nano Res.

[20] Tran HN, Lin CC, Chao HP (2018). Amino acids-intercalated mg/Al layered double hydroxides as dual-electronic adsorbent for effective removal of cationic and oxyanionic metal ions. Sep Purif Technol.

[21] Liu XW, Guo R, Liu H, Yu YQ, Qi XW, Xu JY, Xie CZ (2015). Two series of novel 3D potentially porous heterometallic cu-ln coordination frameworks assembled by 3,4-pyridinedicarboxylic acid with different topologies and channels: syntheses, structures, luminescence and magnetic properties. RSC Adv.

[22] Guo R, You JH, Han F, Liu CB, Zheng GY, Xiao WC, Liu XW (2017). Controlled synthesis, formation mechanism, and carbon oxidation properties of Ho_2_Cu_2_O_5_ nanoplates prepared with a coordination-complex method. Appl Surf Sci.

[23] Sing KSW, Everett DH, Haul RAW, Moscou L, Pierotti RA, Rouquerol J, Siemieniewska T (1985). Reporting physisorption data for gas/solid systems with special reference to the determination of surface area and porosity. Pure Appl Chem.

[24] Tanzifi M, Yaraki MT, Kiadehi AD, Hosseini SH, Olazar M, Bharti AK, Agarwal S, Gupta VK, Kazemi A (2018). Adsorption of Amido Black 10 B from aqueous solution using polyaniline/SiO_2_ nanocomposite: experimental investigation and artificial neural network modeling. J Colloid Interface Sci.

[25] Wagner CD, Riggs WM, Davis LE, Moulder JF, Muilenberg G (1979). Handbook of X-ray photoelectron spectroscopy.

[26] Wagner CD (1990). Practical surface analysis, Vol 1: Auger and X-ray Photoelectron Spectroscopy.

[27] Moulder JF, Stickle WF, Sobol PE, Bomben KD (1992). Handbook of X-ray Photoelectron Spectroscopy.

[28] Liu X, Liu HL, Wen WX, Li XM, Fang N, Wang XH, Wu JH (2015). Facile synthesis and photocatalytic activity of bi-phase dispersible cu-ZnO hybrid nanoparticles. Nanoscale Res Lett.

[29] Miller DJ, Biesinger MC, McIntyre NS (2002). Interactions of CO_2_ and CO at fractional atmosphere pressures with iron and iron oxide surfaces: one possible mechanism for surface contamination. Surf Interface Anal.

[30] You JH, Wang RC, Han F, Guo R, Liu XW (2018). Synthesis and luminescence properties of Mn^3+^, Bi^3+^ co-doped Y_6_WO_12_ for blue phosphor. Rare Metals.

[31] Li L, Qi YH, Lu JR, Lin SL, An WJ, Liang YH, Cui WQ (2016). A stable Ag_3_PO_4_@g-C_3_N_4_ hybrid core@shell composite with enhanced visible light photocatalytic degradation. Appl Catal B Environ.

[32] Foo KY, Hameed BH (2010). Insights into the modeling of adsorption isotherm systems. Chem Eng J.

[33] Atkins P, Paula JD (2015). Atkins’ physical chemistry.

[34] Gobi K, Mashitah MD, Vadivelu VM (2011). Adsorptive removal of methylene blue using novel adsorbent from palm oil mill effluent waste activated sludge: equilibrium, thermodynamics and kinetic studies. Chem Eng J.

[35] Tang H, Zhao Y, Yang XN, Liu DM, Shan SJ, Cui FY, Xing BS (2017). Understanding the pH-dependent adsorption of ionizable compounds on graphene oxide using molecular dynamics simulations. Environ Sci Nano.

[36] Hameed BH, El-Khaiary MI (2008). Equilibrium, kinetics and mechanism of malachite green adsorption on activated carbon prepared from bamboo by K_2_CO_3_ activation and subsequent gasification with CO_2_. J Hazard Mater.

[37] Zhou YM, Zhang M, Hu XY, Wang XH, Niu JY, Ma TS (2013). Adsorption of cationic dyes on a cellulose-based multicarboxyl adsorbent. J Chem Eng Data.

[38] Liu XW, Wang RC, Ni ZY, Zhou WL, Du YC, Ye ZQ, Guo R (2018). Facile synthesis and selective adsorption properties of Sm_2_CuO_4_ for malachite green: kinetics, thermodynamics and DFT studies. J Alloys Compd.

